# The physician as person framework: How human nature impacts empathy, depression, burnout, and the practice of medicine

**Published:** 2017-12-15

**Authors:** Lester Liao

**Affiliations:** 1 Department of Pediatrics, University of Alberta, Alberta, Canada; 2 Arts and Humanities Program in Health and Medicine, University of Alberta, Alberta, Canada

## Abstract

Troubling trends of depression, burnout, and declines in empathy have been demonstrated amongst residents. I argue that while interventions in medical education are helpful, a new perspective on the issue requires a more fundamental understanding of this problem. Rather than training physicians to act in certain ways, we must first recognize that physicians are first and foremost people. This core principle forms the basis of the framework that educators can use to help learners. Five areas of humanity with implications for physicians are discussed: 1) Physicians and patients share their humanity; 2) People are self-integrated in both personal and professional lives; 3) People are dynamic, thoughtful, and emotional; 4) People are finite; and 5) People are moral beings. Recognizing these can mitigate various factors contributing to current struggles. I also discuss practical implications of this framework to help residents flourish.

As the medical world evolves and trainees rely more heavily on technical instruments and an ever increasing knowledge base, unique challenges are presented to the newest ranks of physicians. There are troubling trends in depression and burnout.^1^–^8^ The prevalence of depression/depressive symptoms is estimated to be close to 29% in residents^1^ and 27% in medical students.^6^ Other studies have highlighted concerns with anxiety and psychological distress.^8^–^10^ Empathy has also been in decline, a trend that is often most notable during clinical training years and recognized by learners themselves.^11^–^17^ While there is helpful research surrounding empathy interventions, such as communication skills training^18^–^20^ or mindfulness-based stress reduction training,^21^–^24^ the empathy concerns persist. I propose a conceptual shift in understanding the cause of these issues. Educators must first grasp that physicians experience these problems, not ultimately as physicians, but as people. Reframing the issue this way allows us to address the deeper level of conflict between clinical medicine and human needs, which provides a more robust and informed framework for further skills training.

The heavy pressures of the clinical setting can lead learners to ignore their humanity. As learners neglect their humanity throughout training, the first step in supporting them is to help them remember that they *are* human, not to tell them to *act* more human. This is the basis of the framework of the physician as person: Everyone must live and work within natural human constraints to function well as people. Educators must first become aware and convinced of this central ideological paradigm. Only then can we develop a practice methodology that is conducive to learners flourishing. I propose five elements to consider in this preliminary framework to encourage holistic medical practice.

First, people are people, whether physician or patient. Encounters with patients occur primarily between two people. Secondarily, they hold the roles of doctor and patient. The biopsychosocial model applies to patients *and* physicians. Physicians that have braved illnesses themselves know this well.^25^,^26^ Coming alongside patients is not simply a matter of adopting their perspectives but also identifying the possibility of them in ourselves. Treating the physician as person facilitates treating the patient as person. Recognizing humanity in us all is the foundation of the framework.

Second, people are self-integrated. This means everyone has interconnected personal and professional lives that cannot artificially be separated. Physicians tacitly acknowledge this when they encourage attention to self-care,^27^–^30^ as unhealthy people are unable to be strong physicians. This is reflected similarly in the understanding that personal crises, such as the death of loved ones, impact professional life and may warrant a period of rest. A physician who is caring in both professional and personal contexts is more humane and emotionally healthy than one who only cares at work or at home. Self-integration is basic to healthy human nature.

In contrast, self-compartmentalization and detachment can be harmful. In one example, when facing conflicting reactions towards the struggles of a fellow colleague, residents compartmentalized personal reactions, which included empathy and compassion, away from professional reactions, which focused on duty, training expectations, and becoming professional.^31^ Compartmentalization as a mechanism to cope with negative experiences has been associated with being emotionally labile, unstable low self-esteem, negative mood, and higher anxiety.^32^–^35^ Heavy experiences are a reality in medicine, but rather than having learners suppress their human side, which can lead to callousness, educators must constructively seek ways to alleviate moral and existential burdens. While it is possible for a person to switch gears in different settings, to fully fragment the self into a personal-professional dichotomy is inconsistent with the enduring self that exists across all situations.

Third, people are dynamic, thinking and emotional beings. Narrative medicine and critical reflection amongst learners recognizes this.^36^,^37^ Educators were able to encourage *being* reflective after they recognized that people by nature *are* reflective to various degrees. Physicians are not automatons that dictate options for patients and execute functions.^38^ Physicians who reflect on their own difficult health experiences may also develop greater empathy.^25^,^26^ Similarly, patients at their most vulnerable moments also want personal engagement, interaction, and compassion.^39^ Encouraging such vitality and engagement is a core element of humanistic medicine and the relational nature of empathy.^40^

Fourth, people are finite. They make mistakes and are unable to do everything: this is basic to humanity. If educators can recognize this not only in theory but in praxis, we can change the current culture of fictitious flawlessness. Some pressures are unavoidable and perhaps even conducive to healthy learning, but others based on unrealistic expectations are harmful at both an individual and collective level. Criticisms without plausible solutions and strained team dynamics may result. Mentors and hidden curricula play a major role in either promoting or demoting this mindset.^41^–^43^ Recognizing finitude will shift the perception of professional demands, relieving aforementioned factors related to poor mental health.

Fifth, people are moral beings. Physicians generally act with the moral goal of promoting what they value: health. When they discourage smoking, they are making a moral judgment (e.g., it is good not to smoke). All people have and act according to moral intuitions, whether it is for altruistic or selfish purposes (or both).^44^ Rather than attempting to suppress these intuitions, which can lead to moral distress and division,^45^–^47^ educators must seek ways to build bridges across differences or to openly debate the ethical validity of different practices.^48^,^49^ It is not difficult to imagine that the validity of selfishness will quickly be rejected in an open forum, but other intuitions that seek to honour patients in different ways may need to be further debated. One pediatric intensivist has made multiple suggestions to address moral distress, including acknowledging that moral distress occurs, the need for social relationships, and personal time off.^50^ A grasp of human nature permits and even demands moral discourse.

The development of relevant educational interventions must begin within a framework that has a sound understanding of the human person. The aforementioned interventions show promise because they are built on a holistic understanding of people. Interventions that similarly increase learners’ awareness of their humanity will likely be beneficial. For example, interventions can include readings through literature that powerfully portrays the human condition. Specific learner-driven electives to explore desired topics in health and humanities should also be considered. Interventions must remind learners they are people.

There are, however, limitations to educational interventions. The physician as person framework recognizes that personhood is not easily reducible to one or two qualities. This suggests that a particular targeted intervention will not be sufficient to produce whole scale change. Just as personhood touches all areas of our lives, so must interventions. This means constant exposure through ongoing discussion regarding human nature in medicine, including initiatives such as the International Doctor as a Humanist Project, and not a few educational sessions. It means an ideological shift in the very people that are educators away from prioritizing clear-cut interventions, and instead to embrace ubiquitous discussion and shifts in the hidden curriculum that may be difficult to quantify adequately. This may make change more feasible, as fewer resources (e.g., funds, curricular time) need to be allocated to specific programs. Efforts can instead begin through gradual changes in how educators conduct themselves to positively influence colleagues and learners on a daily basis. Isolated educational interventions are not enough.

This framework also has a major limitation: it is only preliminary. While the overarching framework is to emphasize that learners must operate according to certain basic human qualities to flourish, the number of qualities can be expanded. Similarly, each of the five elements mentioned has much room for further expansion.

Further research should target strong ideological foundations for work in medical education. This would require interdisciplinary perspectives, such as that of philosophy, to further probe questions in basic human nature. It is difficult to directly import the empirical epistemological system of medical treatment into understanding the humanities and medicine without reassessing the underlying theoretical presuppositions. Thus it is crucial to have a comprehensive theory to engage ongoing struggles for medical learners.

Educators and learners must review how we see ourselves as people. The physician as person framework is simply a description of what it means to be a human, and operating within it is to operate according to basic human nature. Only after recognizing this foundation and its implications of self-integration, finitude, emotionality, and morality can downstream issues of depression, burnout, and empathy decline be adequately addressed. Today once more we must remember that the physician is a person.
